# Deciphering the Genetic Inheritance of Tocopherols in Indian Mustard (*Brassica juncea* L. Czern and Coss)

**DOI:** 10.3390/plants11131779

**Published:** 2022-07-05

**Authors:** Vijay Kamal Meena, Yashpal Taak, Rajat Chaudhary, Subhash Chand, Manoj Kumar Patel, Vignesh Muthusamy, Sangita Yadav, Navinder Saini, Sujata Vasudev, Devendra Kumar Yadava

**Affiliations:** 1 Division of Genetics, ICAR—Indian Agricultural Research Institute, New Delhi 110012, India; vjkamal93@gmail.com (V.K.M.); yashpaltaak@gmail.com (Y.T.); rajatbio007@gmail.com (R.C.); subhashchand5415@gmail.com (S.C.); patel.manoj39@gmail.com (M.K.P.); pmvignesh@yahoo.co.in (V.M.); navin12@gmail.com (N.S.); sujatavasudev@gmail.com (S.V.); 2 Division of Seed Science and Technology, ICAR—Indian Agricultural Research Institute, New Delhi 110012, India; sangitaydv@gmail.com

**Keywords:** generation mean analysis, tocopherols, Indian mustard, inheritance, epistatic interaction, seed oil

## Abstract

Tocopherol is vital for the nutritional value and stability of Indian mustard (*Brassica juncea* L. Czern and Coss) oil; nonetheless, the lack of information on genetic control is hampering its improvement. In this study, six populations (P_1_, P_2_, F_1_, F_2_, BC_1_P_1_, and BC_1_P_2_) of RLC3 × NPJ203 were evaluated in a family block design to evaluate the inheritance pattern, gene effects, and various other genetic parameters of tocopherol content (α, γ, and total), using generation mean analysis. The comparison of direct and reciprocal crosses indicated that the tocopherol content was not influenced by maternal inheritance. Negative directional heterosis showed that ATC, GTC, and TTC are governed by recessive genes. Potence ratio and degree of dominance highlighted an over-dominance type of gene interaction for GTC and TTC, whereas ATC was governed by epistatic interactions. Furthermore, the six-parameter model revealed a duplicate gene action for α-tocopherol content. Broad and narrow sense heritability coupled with genetic advances were high.

## 1. Introduction

Tocopherols are essential micronutrients for humans and mammals [1] that are synthesized only by plants, a few cyanobacteria, and green algae [2]. Oilseed crops are the richest source of naturally occurring tocopherols [1]. The most potent fat-soluble antioxidants existing in nature, tocopherols, occur in four naturally occurring forms (α, β, γ, and δ), varying in the number and position of methyl groups on the chromanol ring [2]. The most abundant tocopherols (vitamin E) in *Brassica* oil are α-tocopherol (ATC) and γ-tocopherol (GTC), as well as a small proportion of δ-tocopherol (DTC) [3,4,5]. The ATC is considered to have the highest nutritional value for humans and livestock [6,7]. Owing to its health benefits, improving the composition of vitamin E and increasing the ATC in staple crops, including oilseed crops, has been a major crop breeding objective [8]. Seeds of rapeseed vary widely in terms of their tocopherol content, ranging from 182 to 367 ppm, and tocopherol composition [1]. Fritsche et al. [9] reported an even broader range of variation (197.5 to 460.1 ppm) for total tocopherol content (TTC) in the seeds of *B. napus* germplasm. This prevailing genetic variation incentivizes plant breeders to develop genotypes with an improved tocopherol content; however, the genetic mechanisms underlying this large variation remain unclear.

Tocopherol content is a complex trait governed by several genetic loci [10]. The phenotypic variation in content and composition of tocopherol is primarily due to genetic effects, especially additive gene effects; however, environmental factors also contribute to variations [10]. Goffman et al. [11] described a highly significant general combining ability (GCA) effect for ATC, GTC, and TTC composition; and a significant specific combining ability (SCA) effect for ATC employing diallel analysis in rapeseed. Transgressive segregants and high heritability, ranging from 0.65 to 0.78, were also observed [10]. 

Tocopherol biosynthesis is initiated in the plant’s cytoplasm and finally processed in the plastids [12]. There are seven genes involved in the biosynthesis of tocopherol pathways, and two genes, *VTE3* and *VTE4,* encoding methyltransferase enzymes are responsible for the different methylation patterns on the aromatic ring [2]. The *VTE3* gene encodes for 2-methyl-6-phytyl-1, 4-benzoquinone methyltransferase (MPBQ MT) that catalyzes the methylation of MPBQ, resulting in 2,3-dimethyl-5-phytyl-1, 4-benzoquinone (DMPBQ). This is an important step in the tocopherol synthesis, finally leading to the production of ATC and GTC [10,13]. The *VTE4* gene encoding for γ-tocopherol methyltransferase (gTMT) catalyzes the conversion from δ- to β- and γ- to α-tocopherol [14]. The genes encoding for the key enzymes (*VTE*, loci 1–6; *PDS1*) have been characterized in model plant species *Arabidopsis thaliana* and are highly conserved among other oilseeds [8,15].

Most tocopherol content and composition research has been conducted on *Brassica napus* [9,10,11,16]. In India, Indian mustard is the predominant species of oilseed brassicas, occupying more than 90% area under cultivation [17]. Despite being a predominant crop, the lack of in-depth knowledge of the genetic control of tocopherols has hampered the improvement of the nutritional value and stability features of Indian mustard seed oil. Therefore, in the present study, generation mean analysis was used to understand the inheritance pattern and the gene actions governing tocopherol content for the development of suitable breeding programs to enhance the quality and quantity of tocopherol in Indian mustard. 

## 2. Results and Discussion

### 2.1. The Inheritance Pattern of Tocopherol Contents

The inheritance pattern of ATC, GTC, and TTC was estimated, and a paired *t*-test (Supplementary Table S1) was employed to assess the significance of various means of different tocopherol traits. Only the direct cross (RLC3 × NPJ203) was used further as the reciprocal cross (NPJ203 × RLC3), and its developed populations (F_1_, B_1_, B_2_, and F_2_) were not significantly different from earlier ones (Table 1).

**Figure 1:**
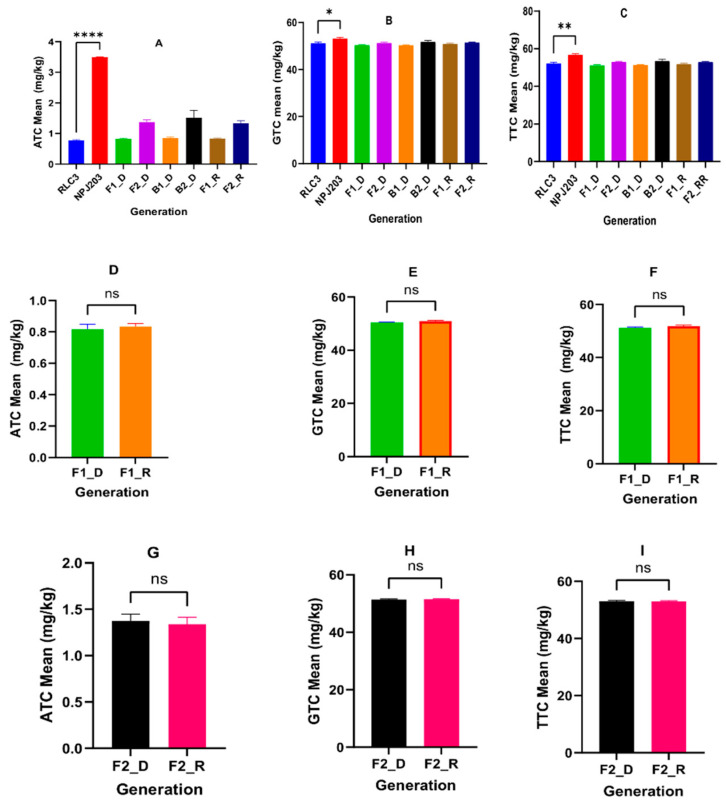
Mean comparison of tocopherol content in various populations developed by crossing between RLC3 and NPJ203 in both directions. Mean comparison of six generations of the direct cross for ATC (**A**), GTC (**B**), and TTC (**C**). Mean comparison of F1s of the direct (F1_D) and reciprocal (F_1__R) crosses for ATC (**D**), GTC (**E**), and TTC (**F**). Mean comparison of F_2_ population of direct (F_2__D) and reciprocal (F_2__R) cross for ATC (**G**), GTC (**H**), and TTC (**I**). * Significant at 5% level of significance (*p* < 0.05); ** highly significant at 1% level of significance (*p* < 0.01); **** highly significant (*p* < 0.0001); ns, non-significant difference.

The tocopherol content in the present study showed no differences in the F_1s_ of direct and reciprocal crosses, clearly suggesting that these are not influenced by maternal inheritance and follow Mendelian inheritance (Figure 1).

**Figure 2:**
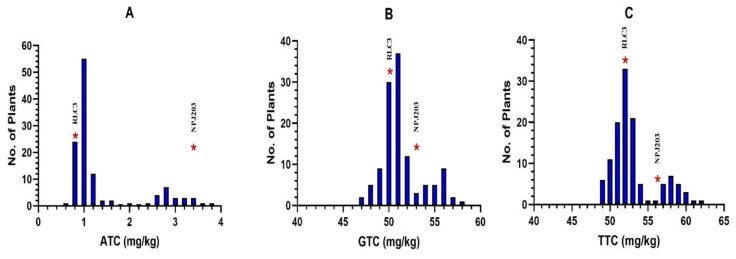
Frequency distribution of tocopherol content of different forms in the F_2_ population developed by RLC3 × NPJ203. Skewed frequency distribution was observed for ATC (**A**), whereas there was a near-normal distribution for GTC (**B**) and TTC (**C**).

Previously, the absence of maternal inheritance for tocopherol content (ATC and GTC) was reported in *B. napus* using diallel analysis [11] and genotype × environment interaction study [3]. For direct cross, parents RLC3 and NPJ203 were statistically significant for mean values of ATC (*p* < 0.001), GTC (*p* < 0.05), and TTC (*p* < 0.01) (Table 1; Supplementary Table S1). The α and γ tocopherol content ratio (AGR) ranged between 1% and 2% in RLC3 and 5–6% in NPJ203. The low AGR value suggested that Indian mustard genotypes are rich in GTC and TTC but possess very low ATC due to the low expression of the *VTE4* (gamma-tocopherol methyltransferase) gene responsible for the conversion of GTC to ATC [18]. To study the nature of the gene’s action for tocopherol content, the means of parents were compared with F_1_ (ATC: 0.815 mg/kg, GTC: 50.418 mg/kg, and TTC: 51.188 mg/kg), and results indicated that F_1_ was significantly different from NPJ203 and non-significant with RLC3 (Figure 1). In BC_1_P_1_, selected and analyzed plants were similar to the RLC3 parent; in BC_1_P_2_, 6 out of 15 plants were NPJ203 type for ATC, GTC, and TTC, and the rest were similar to RLC3. The study, therefore, highlighted that high ATC, GTC, and TTC in Indian mustard are governed by recessive genes. This is the first report on the inheritance pattern of different tocopherol forms in Indian mustard. Previously, the recessive nature of alleles was also reported in *B. carinata* [19] and a single recessive locus *Tph2* for high GTC in sunflower [20]. The frequency distribution of tocopherol content of different forms in the F_2_ population developed by RLC3 × NPJ203 (Figure 2) highlighted a skewed distribution pattern by ATC towards the parent RLC3; however, GTC and TTC showed a near-normal distribution. The higher values for different classes of tocopherol content were reported in *B. juncea* (ATC-51.48, GTC-496.61, and TTC-572 ng/mg of seed) [21]. In our findings, low tocopherol content was observed. The reason may be that the freshly harvested seeds were stored for three months before estimation. The reduction during storage in the seed tocopherols has been reported earlier [22], and seed storage time was negatively associated with tocopherol content in rapeseed. Marzena et al. [23,24] reported a 14.4% reduction in TTC after 18 days of storage in *B. napus*. Similarly, a 35% reduction in ATC and a 65% reduction in TTC were reported in wheat after 35 days of storage [25]. Another study reported a 35% reduction in TTC in walnut [26].

### 2.2. Estimation of Genetic Parameters

The genetic parameters, such as broad-sense heritability (H^2^_b_), narrow-sense heritability (H^2^_n_), genetic advance (G_A_), degree of dominance [h/d], additive variance [D], dominance variance [H], environmental variance [E], and correlation between additive and dominance components [F], were estimated for tocopherol content traits; Table 2.

The prevailing genetic variation between genotypes indicated that direct selection might be effective for the improvement of tocopherol content. Selection efficiency is highly related to the heritability and genetic advance of the trait of interest [27,28]. The broad-sense and narrow-sense heritability were found to be very high for GTC (0.932, 0.938) and TTC (0.949, 0.806). Similarly, broad-sense heritability (0.997) was very high for ATC; however, narrow-sense heritability (0.677) was relatively low. The genetic advance was highest for TTC (6.533), followed by GTC (4.764) and ATC (1.724). The predicted genetic gain under selection depends on heritability and genetic advance rather than heritability alone [29]. The heritability of traits could be influenced by the nature of genetic material, sample size, sampling method, experimental design, linkage effect, and calculation method. The genetic advance refers to the improvement in the mean genotypic value of selected individuals over the parental population and is directly influenced by the genetic variability, selection intensity, and heritability [30,31]. Studied tocopherol traits showed very high broad and narrow-sense heritability (except ATC) and genetic advance, indicating that these traits could be improved by employing direct selection [10]. Marwede et al. [16] earlier reported low broad-sense heritability for ATC (0.23), 0.41 (TTC), and 0.5 (GTC) resulting from significant genotype-by-environment interactions in rapeseed.

The [h/d] ratio calculates the degree of dominance [32,33]. A zero value of the [h/d] ratio represents no dominance, whereas a value equal to one shows complete dominance. Values more than one signify over-dominance, and less than one signifies incomplete dominance. In our findings, the value of the degree of dominance was greater than one for all the studied traits (ATC: 3.10, GTC: 2.219, TTC: 2.702), indicating the over-dominance type of gene action in the inheritance of all studied traits and highlighting that heterozygote has genetic value better than the better parent. Therefore, the preponderance of non-additive genetic control suggested that delaying selection in later generations would be rewarding in improving these traits. A relationship between [D] and [H] over all the loci can be calculated by employing the F value [33,34]. F value equal to zero indicates that all the dominant genes are clustered into the high-performing parent; however, a negative value indicates dominant genes are inculcated into the low-performing parent. In our finding, the F value was negative for all the studied traits (ATC: −0.87, GTC: −4.69, TTC: −10.06), suggesting that dominant genes are present in the low-performing parent, i.e., RLC3. Thus, we conclude that these traits are controlled by recessive genes. The √H/D value was highest for ATC (0.972), followed by TTC (0.595) and GTC (0.576).

The ratio of F/√D × H estimates the magnitude and sign of the direction of dominance. A value close to one indicates that the magnitude and the sign of dominance for all the genes governing the trait of interest are equal, but a value near zero implies that the magnitude and sign of the genes are not equal. Therefore, √H/D is a good estimator of dominance and verifies the average dominance of traits. The F/√H×D values were −0.941, −0.838, and −0.942 for ATC, GTC, and TTC, respectively. For all the studied traits, the ratio of F/√H × D was closed to 1 in the negative direction. Therefore, the ratio √H × D and degree of dominance indicated an over-dominance gene action [33,34].

**Figure 3:**
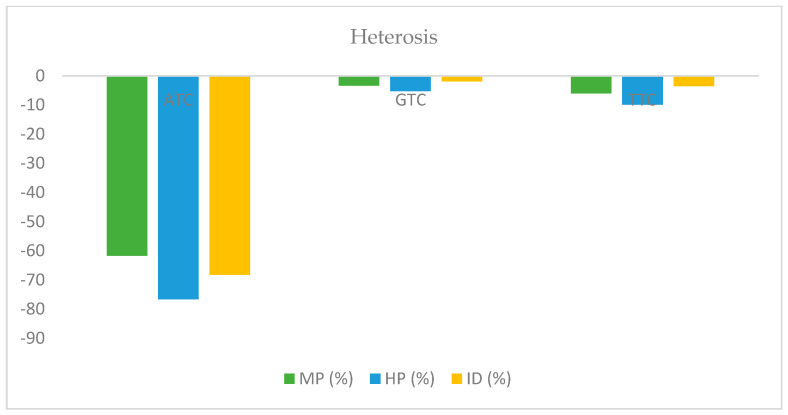
Heterosis and inbreeding depression for all forms of tocopherol content. Mid-parental heterosis (MP), high-parental heterosis (HP), and inbreeding depression (ID) for contents of different tocopherol forms were in the negative direction, suggesting that these traits are governed by recessive genes.

The phenomenon of heterosis and inbreeding depression underlines the type of gene actions that regulate the different quantitative traits, including tocopherol content. The prevailing genetic variation in the population decides the potential use of selection and beneficial utilization in heterosis breeding [28,32]. Heterosis percentage, for instance, for high parent (HP), mid parent (MP), and inbreeding depression (ID), demonstrated that heterosis for all studied tocopherol forms was in the negative direction (Figure 3). The negative direction heterosis for ATC, GTC, and TTC also strongly supports that these traits are governed by recessive genes. Further, selection for high tocopherol traits must be delayed till F_3_ or F_4_ generations to exploit recessive genes and non-segregating lines for the trait of interest. The biparental mating system is very useful for genetic improvement by increasing the genetic recombination frequencies that fasten genetic improvement rates and provide alternative routes to heterosis breeding [34,35].

The potence ratio is principally used to detect the type of dominance [34]. The *p*-value of ±1 denotes complete dominance, values between −1 and +1 represent partial dominance, ≥1 represents over-dominance, and a value of 0 denotes the absence of any type of dominance. The positive and negative signs highlight the direction of the dominance of either parent [36,37]. In our findings, the potence ratio was highest for GTC (−1.749), followed by TTC (−1.403) and ATC (−0.965) (Table 2). The potence ratio for ATC was close to −1, indicating complete dominance, whereas its value surpassed −1 for GTC and TTC, demonstrating an over-dominance type of inheritance. The negative values of the potence ratio sign posted the occurrence of various degrees of recessiveness [38].

### 2.3. Estimation of Scaling Test and Genes Effects

The various scaling tests and gene effects were estimated using means of different generations and are presented in Table 3.

For the absence of non-allelic interactions, the value of A, B, C, and D scaling tests should be equal to zero within the limits of their standard error. Non-allelic interaction is considered a trait if any scale shows a significant difference. Scales C and D provide [l] and [i] gene interaction types, respectively. Furthermore, the [j] type of interaction does not affect C and D, but affects A and B. A and B tests provide information on [i], [j], and [l]-type interactions [36,39]. The six-parameter model was developed by Hayman [40] and Jinks and Jones [41] to estimate genetic components. The significance of [m], [d], [h], [i], [j], and [l] shows the presence of the mean effect, additive effect, dominance effect, additive × additive, additive × dominance and dominance × dominance effects of the gene/s, respectively. All scaling tests were non-significant for the studied traits except scale B, which was statistically significant for ATC. Non-significant scaling tests for GTC and TTC indicate that both traits are controlled by a single gene (intra-allelic interaction). In contrast, the significance of the B test for ATC suggests that the trait is under the influence of epistatic interaction.

Gene effects from the six-parameter model revealed that mean [m] and additive effects [d] were significant for all studied traits. Earlier, tocopherol content was also reported with the predominance of an additive effect in rapeseed [1,3,11,19]. Other gene effects such as dominance effect [h], additive × additive [i], additive × dominance [j], and dominance × dominance [l] were non-significant for GTC and TTC. However, [h], [j], and [l] types of gene effects were significant for ATC. The absence of non-allelic interactions (epistatic interaction) has positive implications for GTC and TTC in their breeding program management. These interactions can impede the identification of suitable genotypes and hinder genetic mapping [42]. For ATC, genetic parameters such as [m], [d], [h], [j], and [l] were significant. The negative sign of [j] suggested the dispersion of genes in both parents [43]. The positive or negative sign of [d] depends on which parent has been chosen as P_1_ [44,45]. Besides, the opposite signs of [h] and [l] were an indicator of the duplicate gene interaction, whereas signs in the same direction indicated complementary interaction [46]. In this study, [h] and [l] had the opposite signs for ATC, indicating duplicate gene action. However, recent reports indicated that ATC is governed by a major gene, i.e., *VTE4*, that converts GTC into ATC. Contemporary studies have shown that the loss of function of *VTE4* has completely replaced ATC with GTC, both in safflower [47] and sunflower [48]. Similarly, increased ATC and reduced GTC has been testified by increasing activity of *VTE4* promoter in soybean [49], insertion of additional copies of *VTE4* in sunflower [20], and overexpression of *VTE4* in other plant species viz., *Arabidopsis*, soybean, and Indian mustard [2,14,21,50].

## 3. Materials and Methods

### 3.1. Selection of Genotypes

Contrasting genotypes (RLC3 and NPJ203) for tocopherol traits were selected as parents and were raised at the experimental farm (latitude 28°38′ N; altitude 77°09′ E and 228.61 m amsl) of the Division of Genetics, ICAR-Indian Agricultural Research Institute (IARI), New Delhi, India, during *rabi* 2017–2018 (Table 4).

### 3.2. Development of Different Breeding Populations

The direct and reciprocal crosses were attempted using RLC3 (P_1_) and NPJ203 (P_2_) genotypes at the experimental farm of the Division of Genetics, ICAR-IARI during *rabi* 2018–2019, and F_1s_ seeds were harvested. The F_2_, BC_1_P_1_ (B_1_: F_1_ backcrossed with RLC3) and BC_1_P_2_ (B_2_; F_1_ backcrossed with NPJ203) populations were developed at ICAR-IARI regional station, Wellington, Tamil Nadu (off-season nursery; 11.37° N, 76.8° E; 1855 m amsl) during *kharif* 2019. The F_2_:F_3_, BC_1_P_1_:F_2_, and BC_1_P_2_:F_2_ were developed by selfing of F_2_, BC_1_P_1_, and BC_1_P_2_ populations, respectively, at the experimental farm of the Division of Genetics, ICAR-IARI during *rabi* 2019–2020.

### 3.3. Experimental Design and Crop Maintenance

The developed six generations (P_1_, P_2_, F_1_, F_2_, B_1_, and B_2_) were raised at the experimental farm of ICAR-IARI, New Delhi, during *rabi* 2020–2021 for the estimation of ATC, GTC, and TTC. The parents, F_1_ and backcross generations (B_1_ and B_2_) were sown in two rows and F_2_ populations in 9 rows, each 5 m in length. The row-to-row and plant-to-plant spacing were kept at 45 cm and 15 cm, respectively. These six generations were replicated twice, employing the family block design. All the recommended agricultural practices, from sowing to harvesting, were followed to raise a healthy crop. Three random plants were selected from each P_1_, P_2_, and F_1_ population, 15 plants each from B_1_ and B_2_, and 120 plants from the F_2_ population of each replication were harvested individually and were used for the estimation of tocopherol traits.

### 3.4. Estimation of Tocopherol Contents

The seeds’ ATC, GTC, and DTC were estimated using high-performance liquid chromatography (HPLC) [9,51,52,53]. Freshly harvested seeds were stored for three months at 4 °C before analyses; 50 mg seeds were ground in 1500 μL n-heptane to extract tocopherols. Further, the solution was incubated at −20 °C for 2 h, and 20 μL was used for HPLC analysis. A silica gel column (5 μM LiChrospher^®^ Si 60, Merck, Darmstadt, Germany) was used to extract tocopherols employing a mobile phase consisting of an n-heptane/isopropanol-mixture (99 + 1; *v* + *v*). The fluorescence detection (excitation at λ = 290 nm and emission at λ = 328 nm) method was used for tocopherol quantification. The retention times were compared with Merck’s tocopherol kit (Merck, Darmstadt, Germany) to identify specific tocopherol forms (DTC: 7.9 min; GTC: 8.9 min; BTC: 9.9 min, and ATC: 10.6 min), and the concentration of single forms was correlated with signal output to calibrate each tocopherol form (Supplementary Figures S1–S11). Additionally, a linear calibration range was obtained for the concentration of the analyzed samples under this study. However, the concentration of β-tocopherol was the lowest; hence, it was not analyzed in this study further. The TTC was estimated by summing up ATC, GTC, and DTC, whereas the tocopherol composition was expressed as the ratio of ATC and GTC, i.e., AGR.

### 3.5. Statistical Analysis

#### 3.5.1. Estimation of Scaling Test

To study gene effects, a scaling test was used to assess the adequacy of an additive-dominance model [54]. As suggested by Singh and Chaudhary [55], four scales (A, B, C, and D) were used to obtain information on the presence or absence of allelic interactions. Scales A and D denote the presence of additive × additive [i] gene action, whereas scales B and C represent additive × dominance [j] and dominance × dominance [l] gene interactions, respectively. In addition, scaling tests were calculated with the following equations:

(1) $$A=2\bar{B_{1}}-\bar{P_{1}}-\bar{F_{1}}$$

(2) $$B=2\bar{B_{2}}-\bar{P_{2}}-\bar{F_{1}}\;\;$$

(3) $$C=4\bar{F_{2}}-2\bar{F_{1}}-\bar{P_{1}}-\bar{P_{2}}\;$$

(4) $$D=2\bar{F_{2}}-\bar{B_{1}}-\bar{B_{2}}$$

where $\bar{P_{1}},\;\bar{P_{2\;}},\;\bar{F_{1}},\;\bar{F_{2}},\;\bar{B_{1}},\;\mathrm{and}\;\bar{B_{2}}$ represent the mean value of *P*_1_, *P*_2_, *F*_1_, *F*_2_, *B*_1_, and *B*_2_, respectively. If any of the scales were significant (non-zero), it indicates the presence of non-allelic interaction (epistasis) and vice versa [39,54]. Furthermore, the three-parameter model [41] is used for the estimation of gene effects if the additive–dominance model is acceptable (absence of non-allelic interactions); however, non-adequacy (presence of non-allelic interactions) results in the use of a six-parameter model [40,56].

#### 3.5.2. Components of Generation Means

In compliance with the additive–dominance model, the mean effect [m], additive effect [d], and dominance effect [h] could be calculated with the following equations [41,54]:

(5) $$\left[m\right]=\frac{1}{2}\left(\bar{P_{1}}\right)+\frac{1}{2}\;\left(\bar{P_{2}}\right)+4\left(\bar{F_{2}}\right)-2\left(\bar{B_{1}}\right)-2\left(\bar{B_{2}}\right)$$

(6) $$\left[d\right]=\frac{1}{2}\left(\bar{P_{1}}\right)-\frac{1}{2}\left(\bar{P_{2}}\right)$$

(7) $$\left[h\right]=6\left(\bar{B_{1}}\right)+6\left(\bar{B_{2}}\right)-8\left(\bar{F_{2}}\right)\;–\left(\bar{F_{1}}\right)-\frac{3}{2}\left(\bar{P_{1}}\right)-\frac{3}{2}\left(\bar{P_{2}}\right)$$

However, interactions were calculated separately in the presence of epistatic effects, and the following equations were used for the analysis of each gene effect employing the mean value of each generation [41]:

(8) $$\left[m\right]=\bar{F_{2}}$$

(9) $$\left[d\right]=\bar{B_{1}}-\bar{B_{2}}$$

(10) $$\left[i\right]=2\left(\bar{B_{1}}\right)+2\left(\bar{B_{2}}\right)-4\left(\bar{F_{2}}\right)$$

(11) $$\left[j\right]=\left(\bar{B_{1}}\right)-\frac{1}{2}\left(\bar{P_{1}}\right)-\left(\bar{B_{2}}\right)+\frac{1}{2}\;\bar{(P_{2}})$$

(12) $$\;\left[l\right]=\left(\bar{P_{1}}\right)+\left(\bar{P_{2}}\right)+2\left(\bar{F_{1}}\right)+4\bar{\left(F_{2}\right)}-4\left(\bar{B_{1}}\right)-4\left(\bar{B_{2}}\right)$$

where [m], [d], [h], [i], [j], and [l] represented mean effect, additive gene effect, dominance gene effect, additive × additive, additive × dominance, and dominance × dominance gene effects, respectively.

#### 3.5.3. Estimation of Various Genetic Parameters

The different components of variation in six populations were analyzed as per Mather and Jinks [29], and the following formulae were used based on *F*_2_ variances:

(13) $$E=\frac{1}{3}\left(VP_{1}+VP_{2}+VF_{1}\right)$$

(14) $$D=4\left(VF_{2}\right)-2\left(VB_{1}+VB_{2}\right)$$

(15) $$H=4\;\left(VF_{2}-\;\frac{1}{2}\left(V_{D}\right)-(V_{E})\right)$$

(16) $$\;F\;=VB_{1}-VB_{2}$$

where E, D, H, and F represented an environmental component of variance, additive genetic variance, dominance genetic variance, and correlation between D and H, overall loci, respectively. Broad-sense (H^2^_b_) and narrow-sense (H^2^_n_) heritability were calculated by employing the following formulae [57]:

(17) $$H_{b}^{2}=\frac{\left[VF_{2}-\frac{VP_{1}+VP_{2}+VF_{1}}{3}\right]}{VF_{2}}$$

(18) $$H_{n}^{2}=\frac{\left[2\;\left(VF_{2}\right)-\left(VB_{1}+VB_{2}\right)\right]}{VF_{2}}\;$$

Genetic advance (G_A_) was calculated as per Johnson et al. [27] by employing the following formula:

(19) $$G_{A}=i.H_{b}^{2}.\sqrt{VF2}$$

where selection intensity was *i* = 5% for the studied traits. Potence ratio (P) was calculated to determine the degree of dominance employing the following formula [37]:

(20) $$P=\left[\frac{\bar{F_{1}}-MP}{\frac{1}{2}\left(\bar{P_{2}}-\bar{P_{1}}\right)}\right]\times 100$$

where P is the relative potence ratio of the gene set; *F*_1_ is the first-generation mean; *P*_1_ is the mean of the lower parent; *P*_2_ is the mean of the higher parent; and *MP* is the mid-parental value [(*P*_1_ + *P*_2_)/2].

## 4. Conclusions

Although the tocopherol biosynthesis pathway has been elucidated in model organisms such as *Arabidopsis thaliana* and *Synechocystis* sp. PCC6803, information on tocopherol inheritance in Indian mustard is inadequate. The present investigation highlighted that tocopherol content and composition are controlled by recessive nuclear genes and are not affected by maternal effects in seeds of Indian mustard. High heritability coupled with genetic advances for the studied traits indicated that direct selection could improve these traits. The potence ratio and degree of dominance highlighted that GTC and TTC showed an over-dominance gene interaction, whereas ATC showed an epistatic interaction. This is the first study on Indian mustard that elucidated the nature of inheritance and gene effects of tocopherol traits employing generation mean analysis. This study has potential applications for enhancing tocopherol content in the seed oil of Indian mustard for enhancing oil quality by incorporating genes, especially for ATC, into exiting elite lines.

## Figures and Tables

**Table 1:** Comparison of means of tocopherol content between various generations of the direct (RLC3 × NPJ203) and the reciprocal (NPJ203 × RLC3) crosses. Where, ATC, GTC, TTC (mean ± standard error) were presented in mg/kg, whereas AGR (ATC/GTC Ratio) in percentage.

Generations	RLC3 × NPJ203 (Direct Cross)				NPJ203 × RLC3 (Reciprocal Cross)			
	ATC	GTC	TTC	AGR%	ATC	GTC	TTC	AGR%
RLC3 (P_1_)	0.768 ± 0.035	51.182 ± 0.515	52.138 ± 0.539	1.50	0.788 ± 0.039	51.097 ± 0.346	52.037 ± 0.363	1.54
NPJ203 (P_2_)	3.492 ± 0.008	53.223 ± 0.425	56.848 ± 0.432	6.56	3.327 ± 0.094	54.47 ± 0.372	57.947 ± 0.414	6.11
F_1_	0.815 ± 0.031	50.418 ± 0.174	51.188 ± 0.293	1.62	0.833 ± 0.035	50.903 ± 0.358	51.883 ± 0.363	1.64
F_2_	1.371 ± 0.077	51.396 ± 0.241	53.03 ± 0.304	2.67	1.338 ± 0.077	51.48 ± 0.205	52.979 ± 0.272	2.60
B_1_	0.853 ± 0.045	50.366 ± 0.302	51.373 ± 0.328	1.69	1.626 ± 0.285	52.527 ± 0.655	54.287 ± 0.911	1.72
B_2_	1.511 ± 0.245	51.766 ± 0.636	53.486 ± 0.882	2.92	0.863 ± 0.047	50.278 ± 0.243	51.282 ± 0.266	3.10

**Table 2:** Genetic parameters and components of variation for tocopherol content of different forms in RLC3 × NPJ203 cross.

Character ^$^	Genetic Parameters and Variation Components ^#^										
	H^2^_b_	H^2^_n_	G_A_	[h/d]	[E]	[D]	[H]	[F]	√H/D	F/√H×D	[P]
ATC	0.997	0.677	1.724	3.140	0.002	0.952	0.899	−0.87	0.972	−0.941	−0.965
GTC	0.932	0.938	4.764	2.219	0.476	9.714	3.227	−4.69	0.576	−0.838	−1.749
TTC	0.949	0.806	6.533	2.702	0.563	17.946	6.360	−10.06	0.595	−0.942	−1.403

^#^ H^2^_b_: broad-sense heritability, H^2^_n_: narrow-sense heritability, G_A_: genetic advance, [h/d]: degree of dominance, [D]: additive variance, [H]: dominance variance, [E]: environmental variance, [F]: correlation between additive and dominance components, [P]: potence ratio. ^$^ ATC: alpha-tocopherol content, GTC: Gamma tocopherol content, TTC: total tocopherol content. Where h/d, D, H, E, and F denote the degree of dominance, additive, dominance, environmental components, and an indicator of correlation between D and H over all loci, respectively.

**Table 3:** Estimates of scaling test and types of gene action using generation means of tocopherol content traits in the cross RLC3 × NPJ203.

Character	Scaling Test				Gene Effects by Six Parameter Model						
	A	B	C	D	[m]	[d]	[h]	[i]	[j]	[l]	Epistasis
**ATC**	−0.122	1.284 **	0.408	0.377	1.371 **	−0.659 **	−2.069 **	−0.754	1.406 **	1.916 **	Duplicate
**GTC**	0.869	0.11	−0.343	0.661	51.396 **	−1.4 **	−3.106	−1.321	−0.759	2.3	Absent
**TTC**	0.581	1.065	−0.758	1.202	53.03 **	−2.113 **	−5.709	−2.404	0.485	4.05	Absent

Where [m], [d], [h], [i], [j], and [l] denote; mean, additive effect, dominance effect, additive × additive, additive × dominance, and dominance × dominance, respectively. ** Significant at 1%.

**Table 4:** Details of the parents used in the estimation of tocopherol content traits.

S.N.	Genotypes	Pedigree/Source	Origin	Tocopherol Content Traits (mg/kg)				
				ATC	GTC	DTC	AGR	TTC
1	RLC3	JM06003 × JM06020	PAU, Ludhiana, Indian	0.74	51.17	0.19	0.01	52.10
2	NPJ203	(EJ9913 × SEJ8) × Laxmi	IARI, New Delhi, Indian	3.49	53.14	0.15	0.07	56.78

RLC3 is a canola-quality variety; NPJ203 is an elite breeding line of Indian mustard.

## Data Availability

All the data are included within the manuscript and the supplementary table.

## Supplementary Materials

The following supporting information can be downloaded at: https://www.mdpi.com/article/10.3390/plants11131779/s1. Table S1: Paired *t*-test between different breeding generations for tocopherol content traits.
