# Supplementary material for: Deciphering the Genetic Inheritance of Tocopherols in Indian Mustard (Brassica juncea L. Czern and Coss)
Source: Plants (Basel). 2022 Jul 5;11(13):1779. doi: 10.3390/plants11131779 (PMC9269207; doi:10.3390/plants11131779)

Supplementary Figure S1: HPLC chromatogram for Tocopherol standards

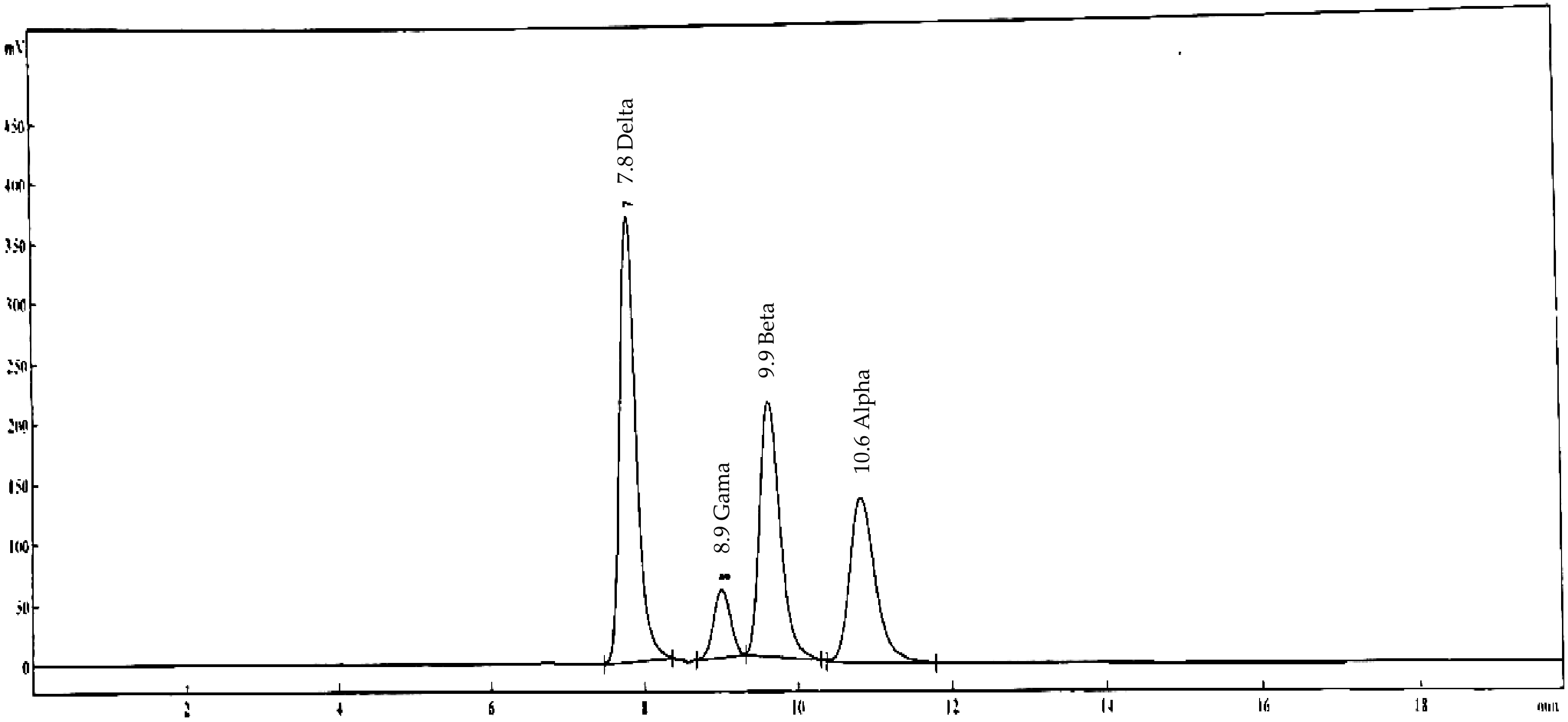

Supplementary Figure S2: HPLC chromatogram for Parent NPJ203

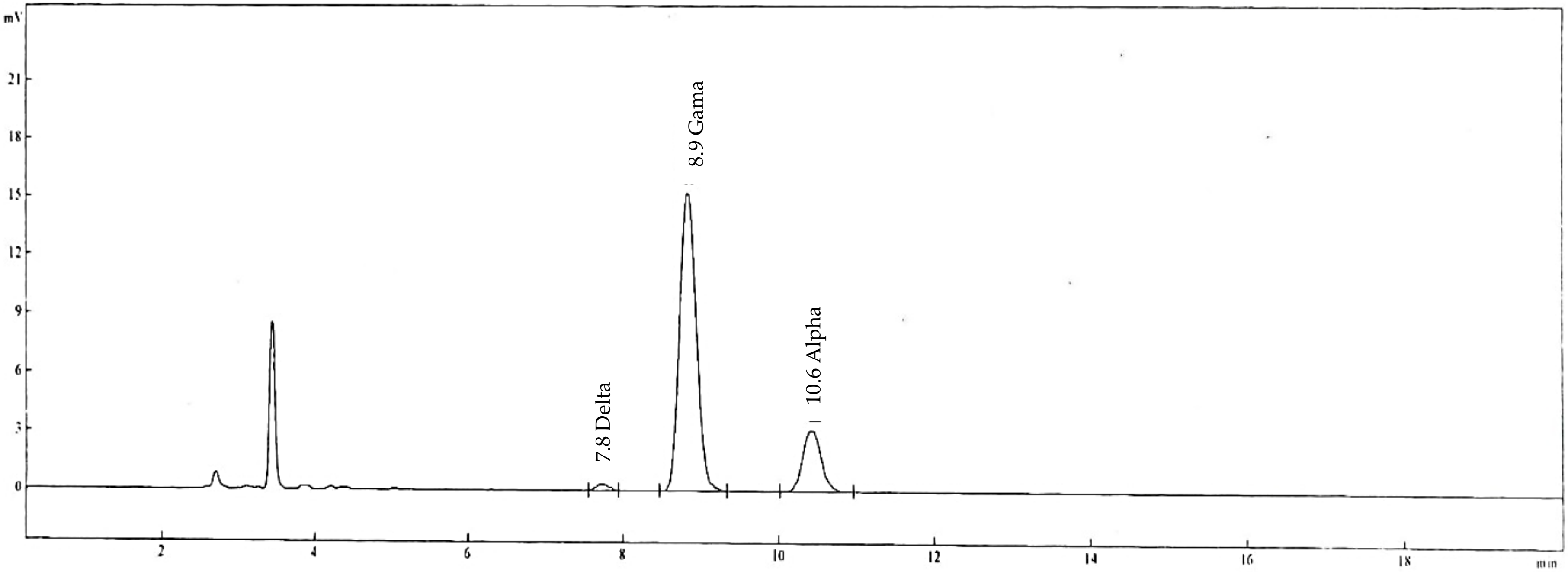

Supplementary Figure S3: HPLC chromatogram for Parent RLC3

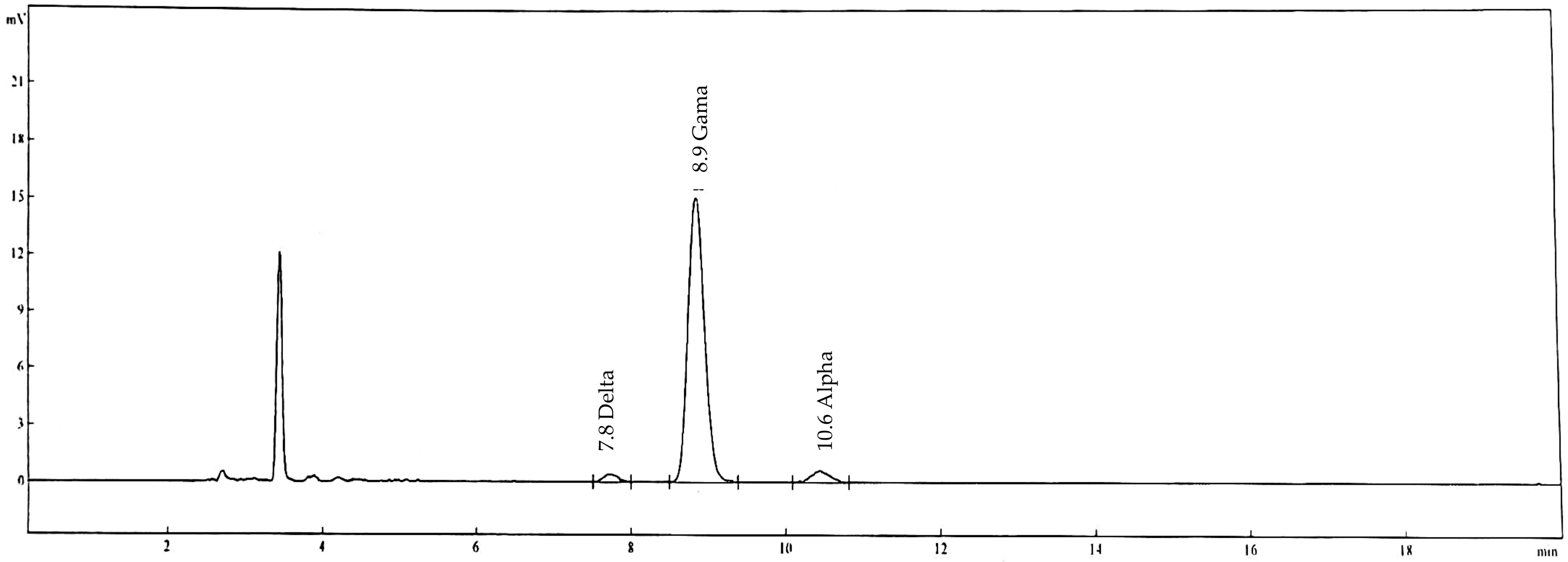

Supplementary Figure S4: HPLC chromatogram for F_1 from cross (RLC3 × NPJ203)

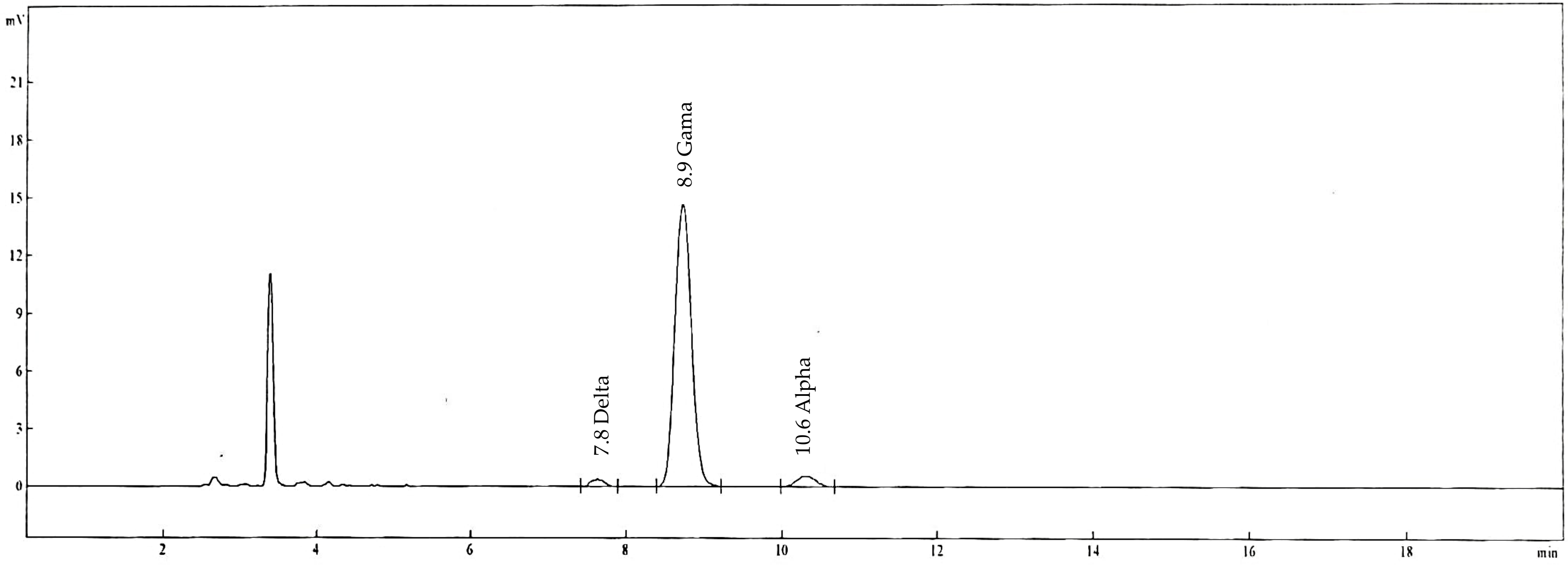

Supplementary Figure S5: HPLC chromatogram for F_1 from cross (NPJ203 × RLC3)

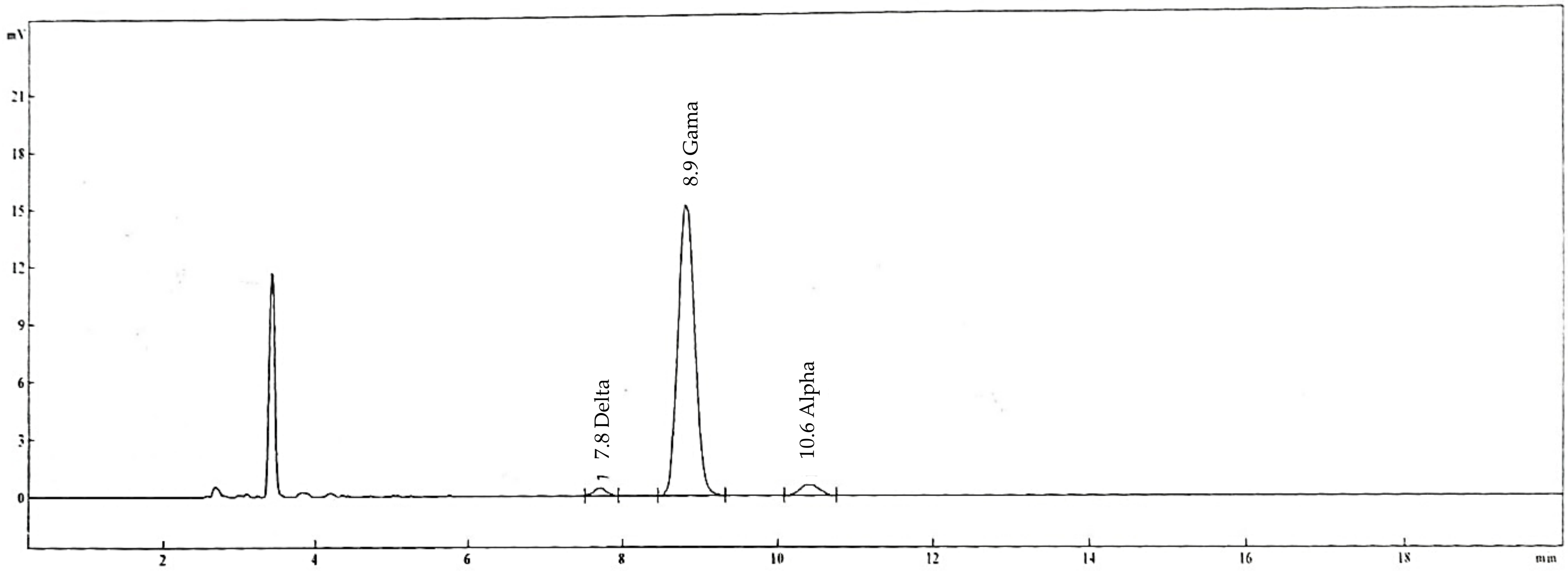

Supplementary Figure S6: HPLC chromatogram for F_2 from cross (RLC3 × NPJ203)

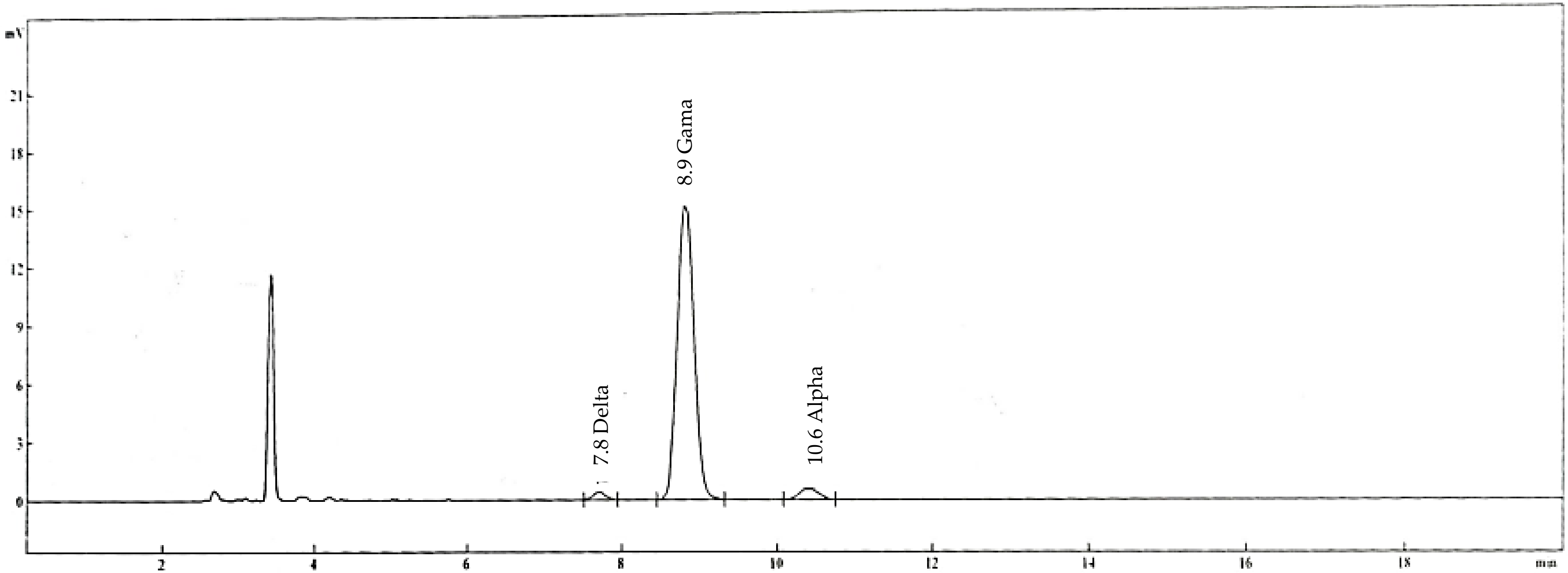

Supplementary Figure S7: HPLC chromatogram for F_2 from cross (NPJ203 × RLC3)

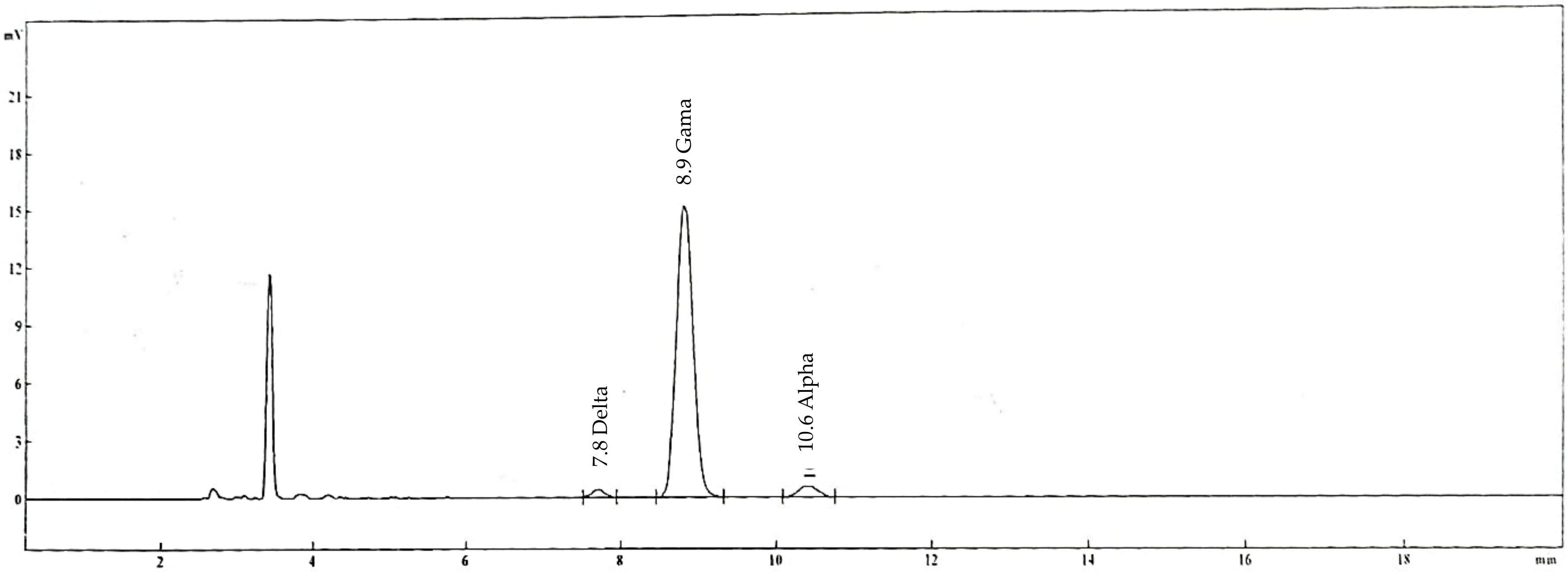

Supplementary Figure S8: HPLC chromatogram for BC1F1 with RLC3 from cross (RLC3 × NPJ203)

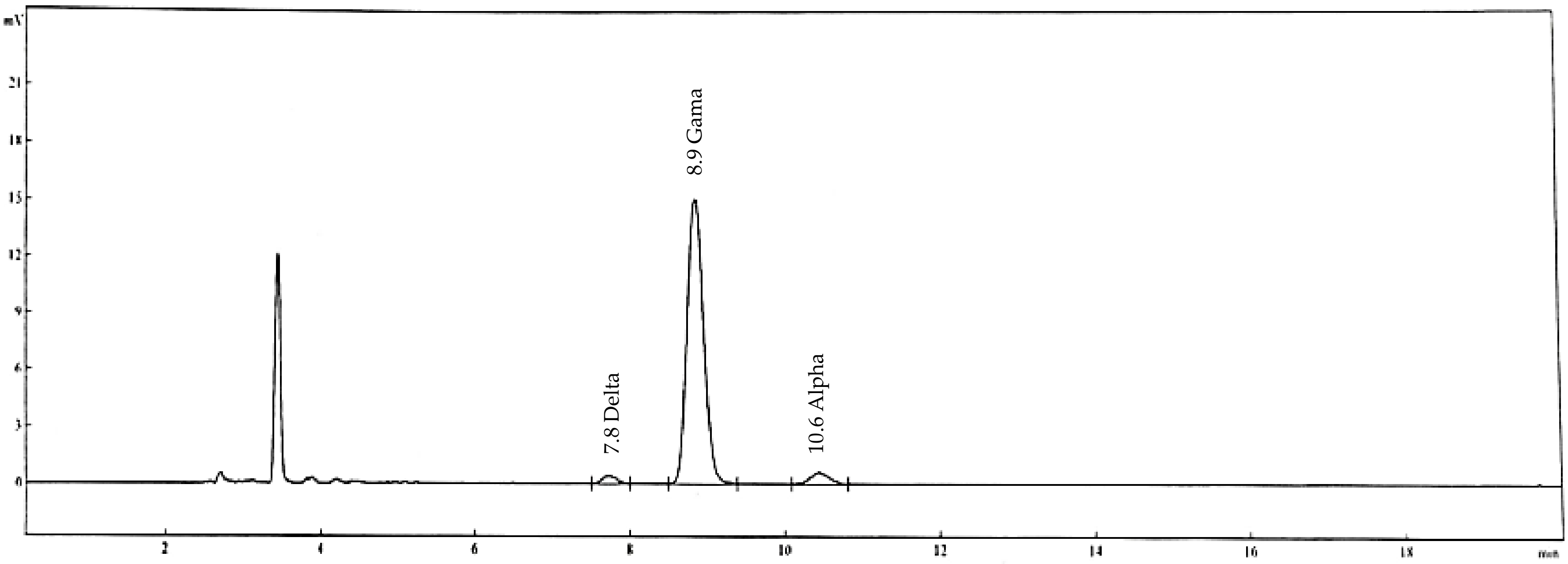

Supplementary Figure S9: HPLC chromatogram for BC1F1 with NPJ203 from cross (RLC3 × NPJ203)

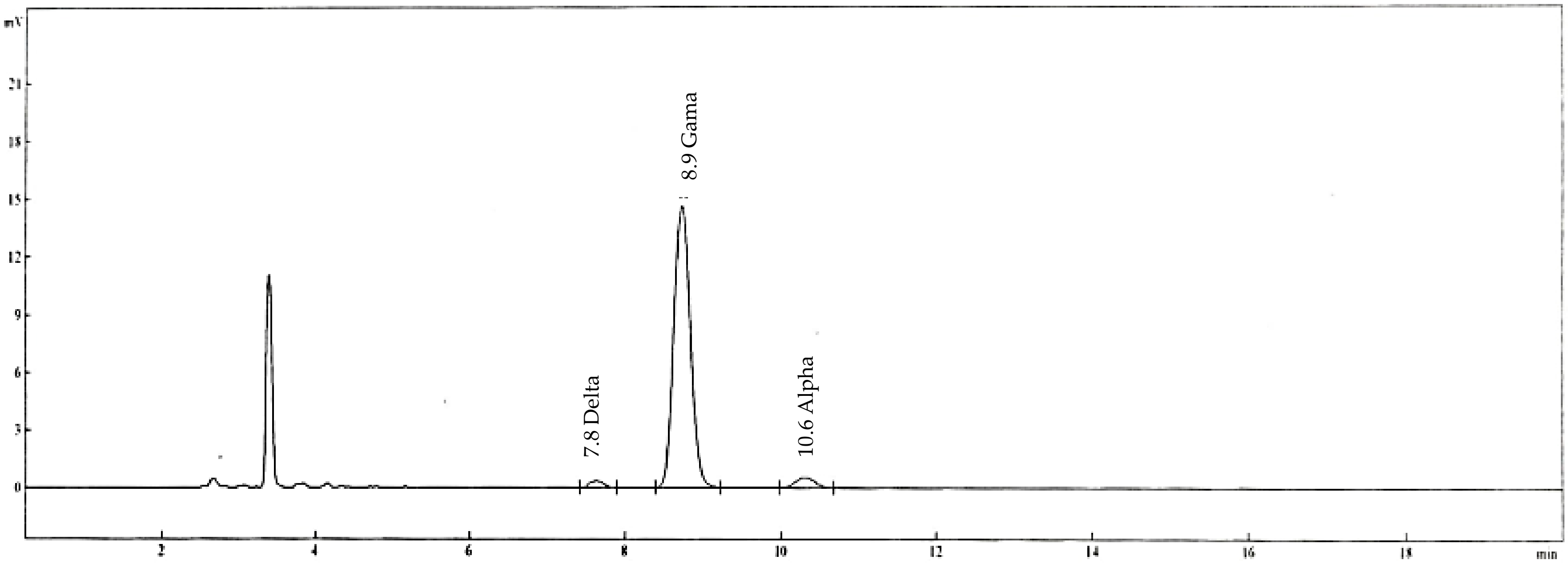

Supplementary Figure S10: HPLC chromatogram for BC1F1 with NPJ203 from cross (NPJ203 × RLC3)

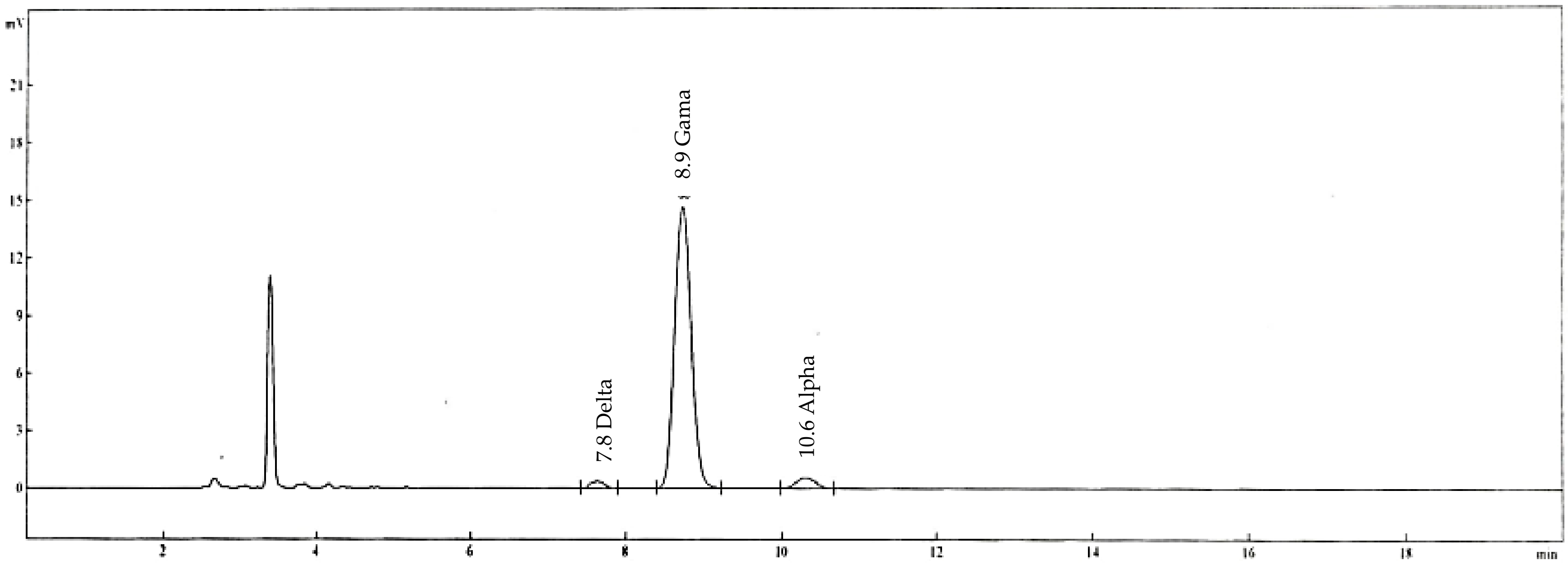

Supplementary Figure S11: HPLC chromatogram for BC1F1 with RLC3 from cross (NPJ203 × RLC3)

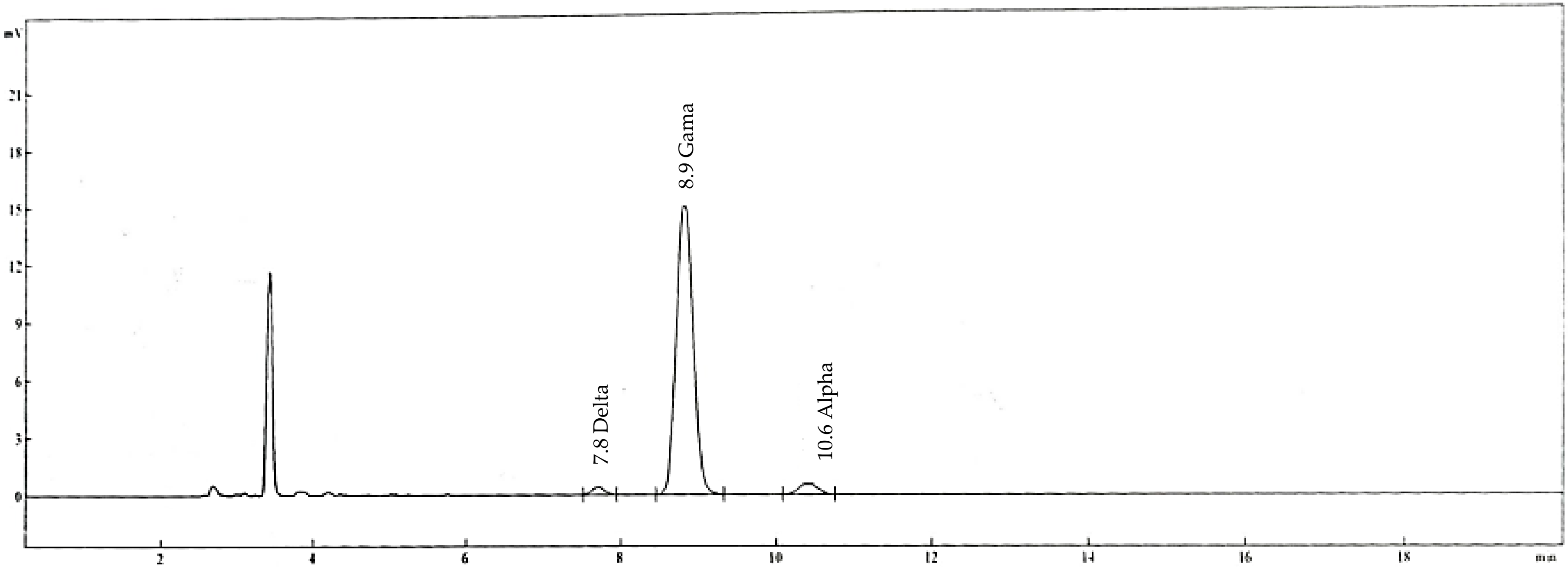

Supplement: Supplementary file 1 [file plants-11-01779-s001.zip › Meena et al 1648712_supplementary_figures_.pdf]
