# Supplementary material for: Deciphering the Genetic Inheritance of Tocopherols in Indian Mustard (Brassica juncea L. Czern and Coss)
Source: Plants (Basel). 2022 Jul 5;11(13):1779. doi: 10.3390/plants11131779 (PMC9269207; doi:10.3390/plants11131779)
Supplement: Supplementary file 1 [file plants-11-01779-s001.zip › Supplementary Table S1.pdf]

Supplementary Table S1: Paired *t*-test between different breeding generations for tocopherol content traits

	ATC		GTC		TTC	
	***p*-value**	***t*-value**	***p*-value**	***t*-value**	***p*-value**	***t*-value**
**RLC3_NPJ203**	0.0002 **	72.50	0.0453 *	4.538	0.0104 **	9.718
**F1D_RLC3**	0.5318 ns	0.7494	0.411 ns	1.031	0.3946 ns	1.076
**F1D_NPJ203**	0.0001 **	90.34	0.0390 *	4.916	0.0108 **	9.523
**F1D_F1R**	0.4045 ns	1.048	0.3889 ns	1.092	0.2519 ns	1.594
**F2D_F2R**	0.7216 ns	0.3571	0.7573 ns	0.3097	0.8901 ns	0.1386

* Significant at 5% level of significance ($p<0.05$); ** highly significant at 1% level of significance ($p<0.01$); and ns-non significant difference.
